# Supplementary material for: Impact of COVID-19 on unmet needs for healthcare in Peru: an interrupted time series analysis
Source: PLOS Glob Public Health. 2025 Oct 10;5(10):e0005036. doi: 10.1371/journal.pgph.0005036 (PMC12513646; doi:10.1371/journal.pgph.0005036)
Supplement: S1 Fig — (PDF) [file pgph.0005036.s001.pdf]

S1 Fig. Interrupted time series analysis of the impact of COVID-19 lockdown on the presence of a health problem in last 4 weeks, stratified by sociodemographic variables.

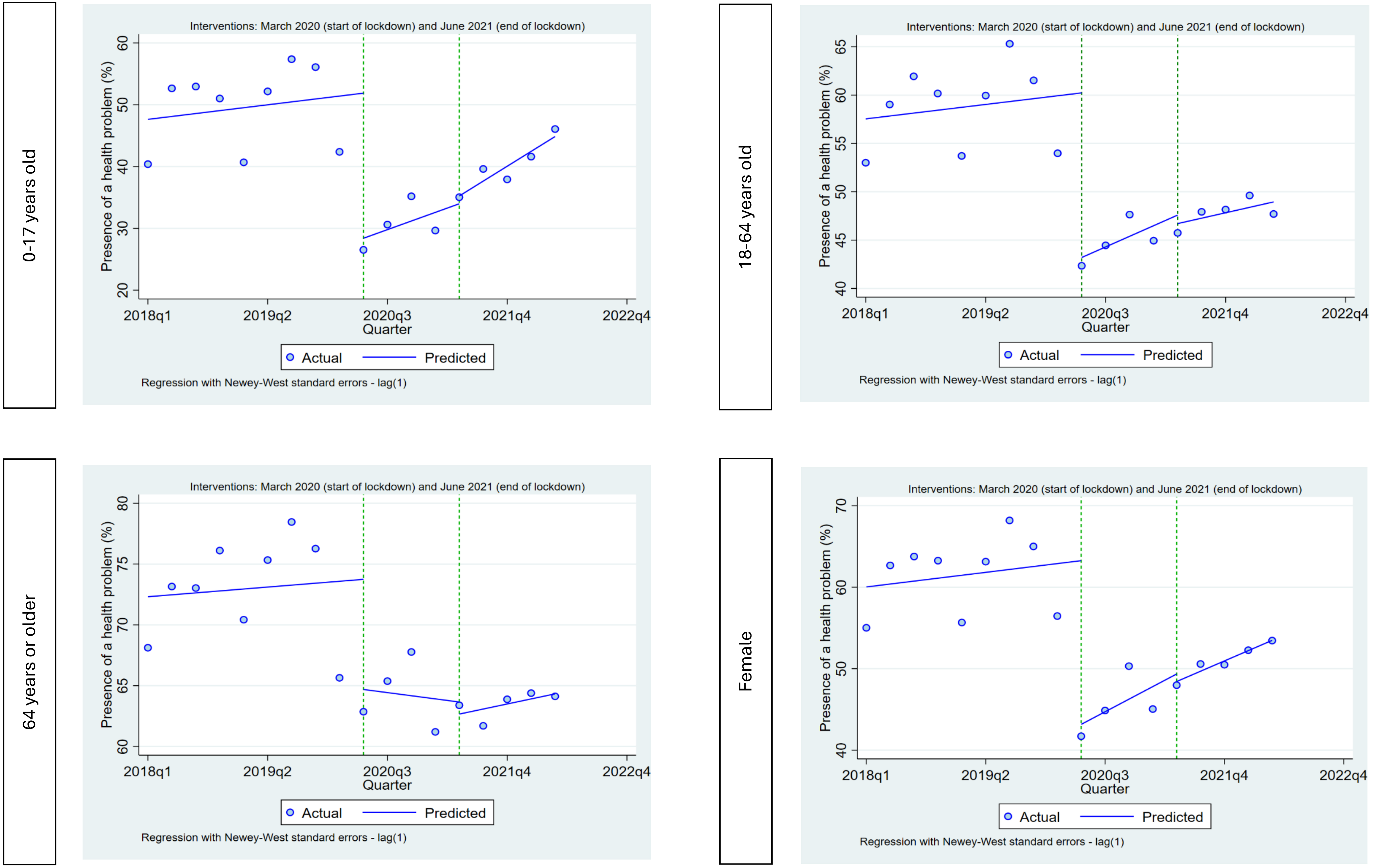

Male

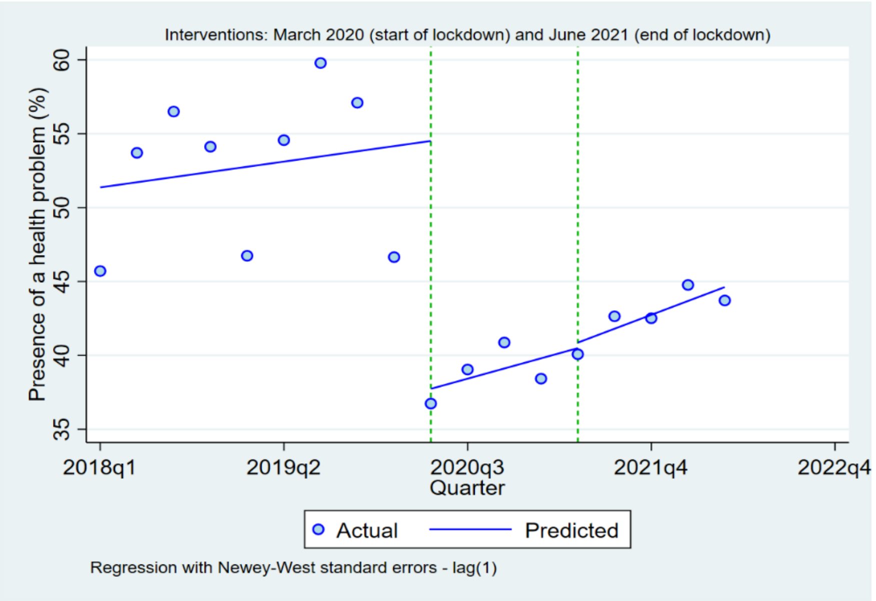

Non-native

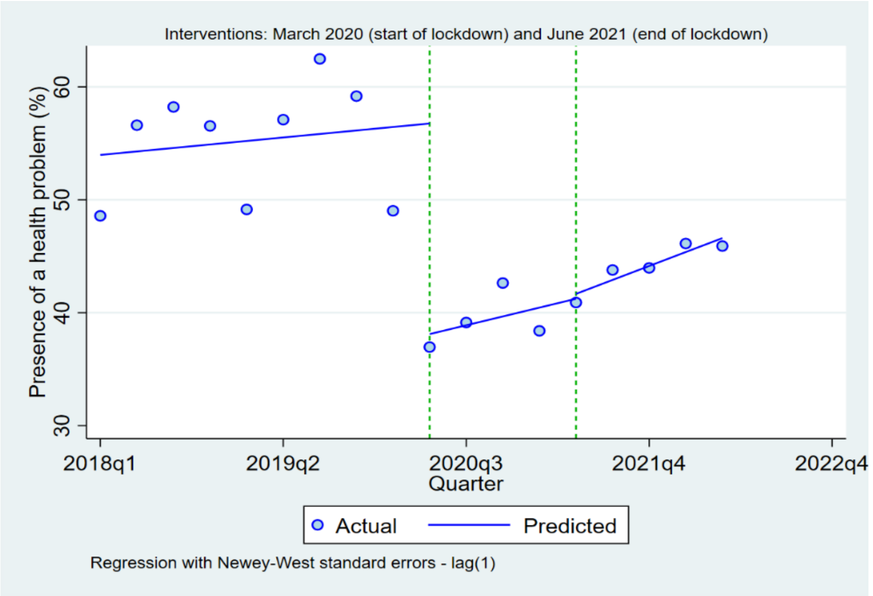

Native

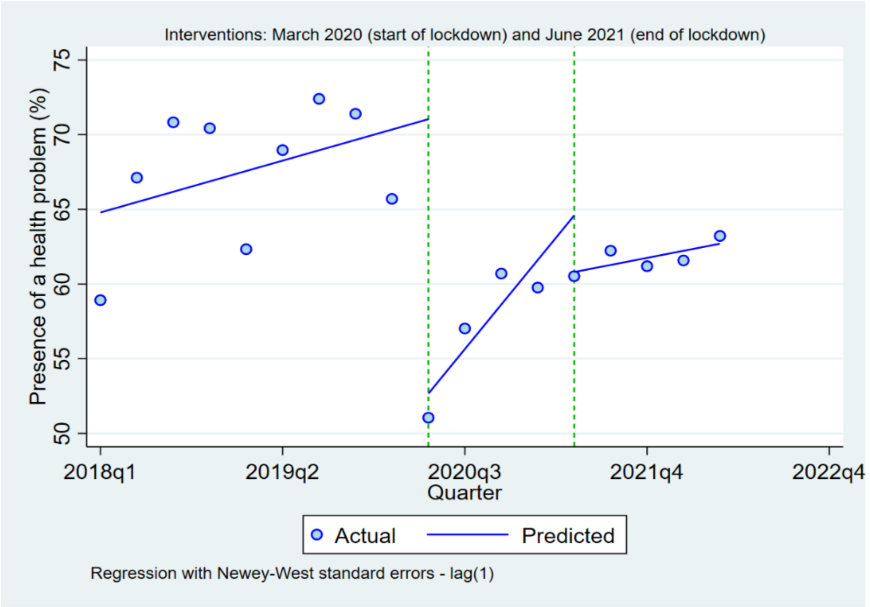

No health insurance

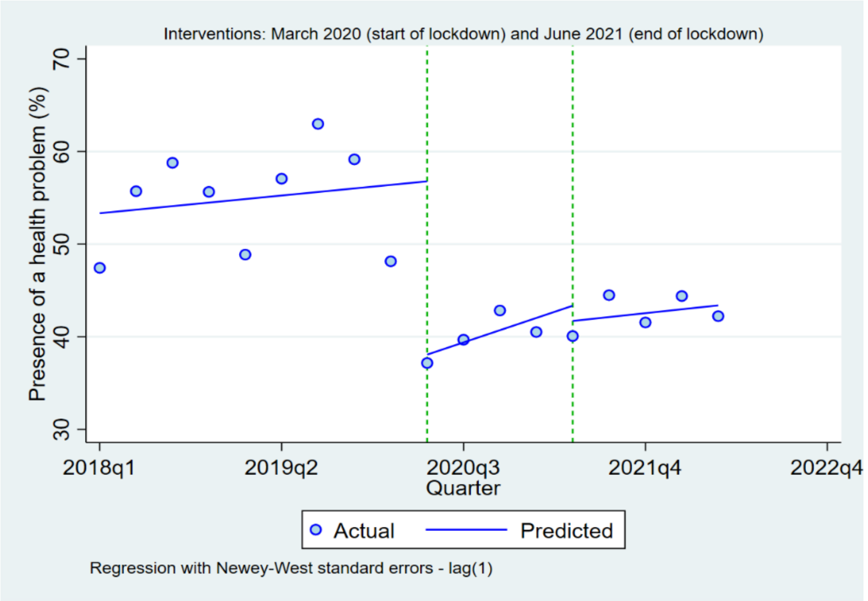

Health insurance

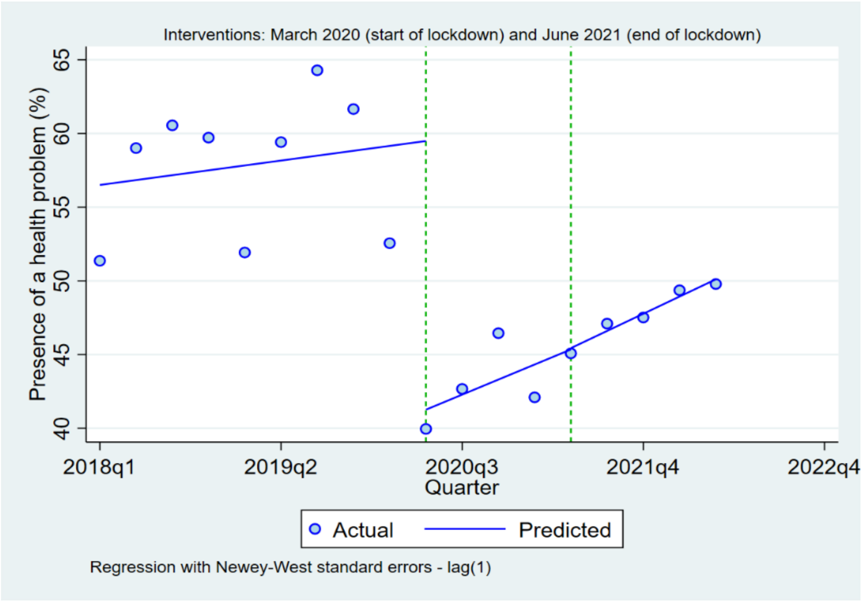

People without disabilities

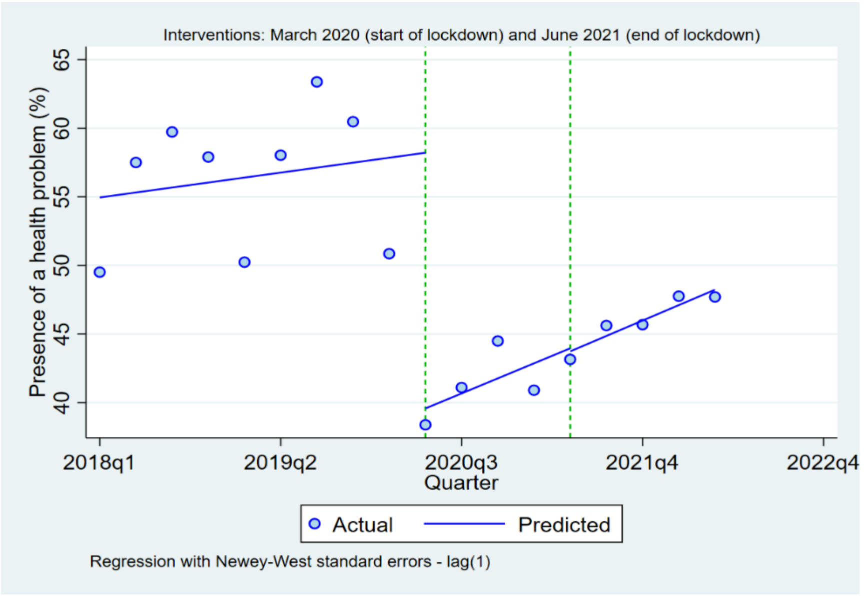

People with disabilities

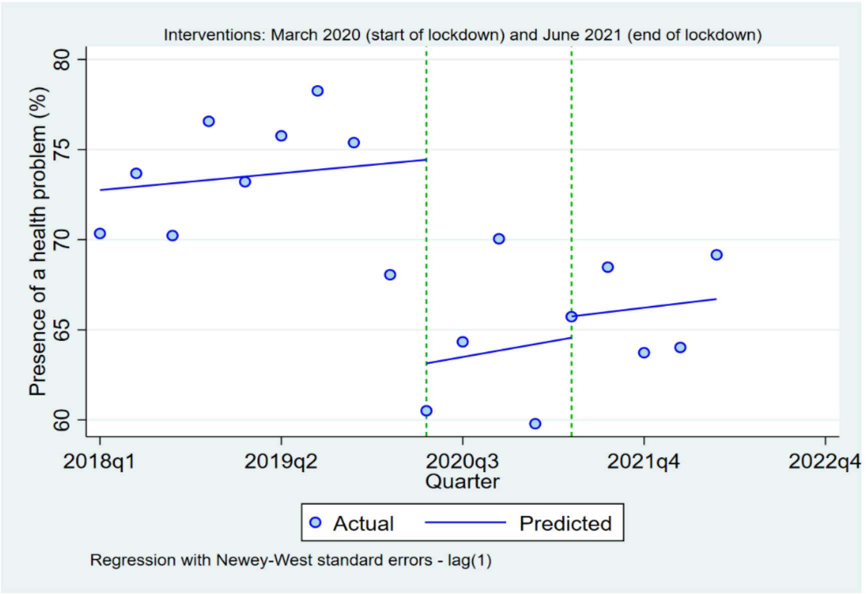

Rural

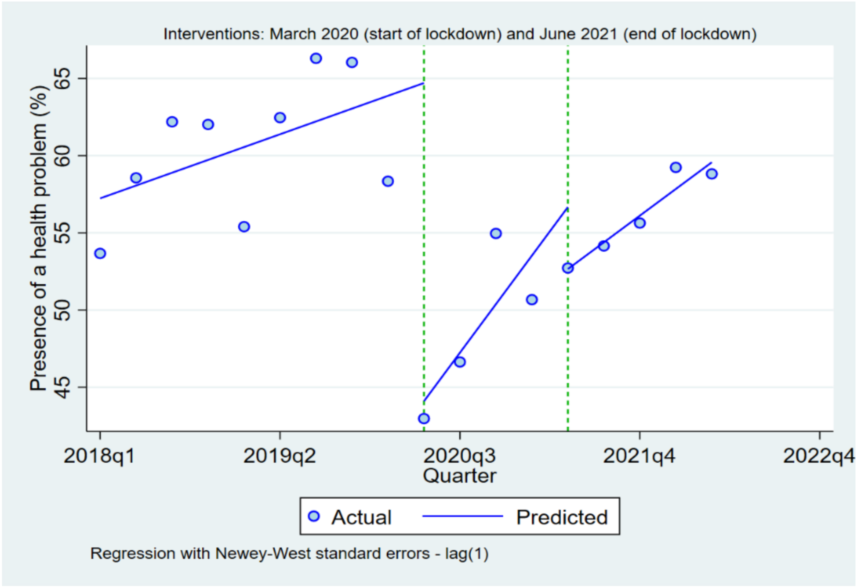

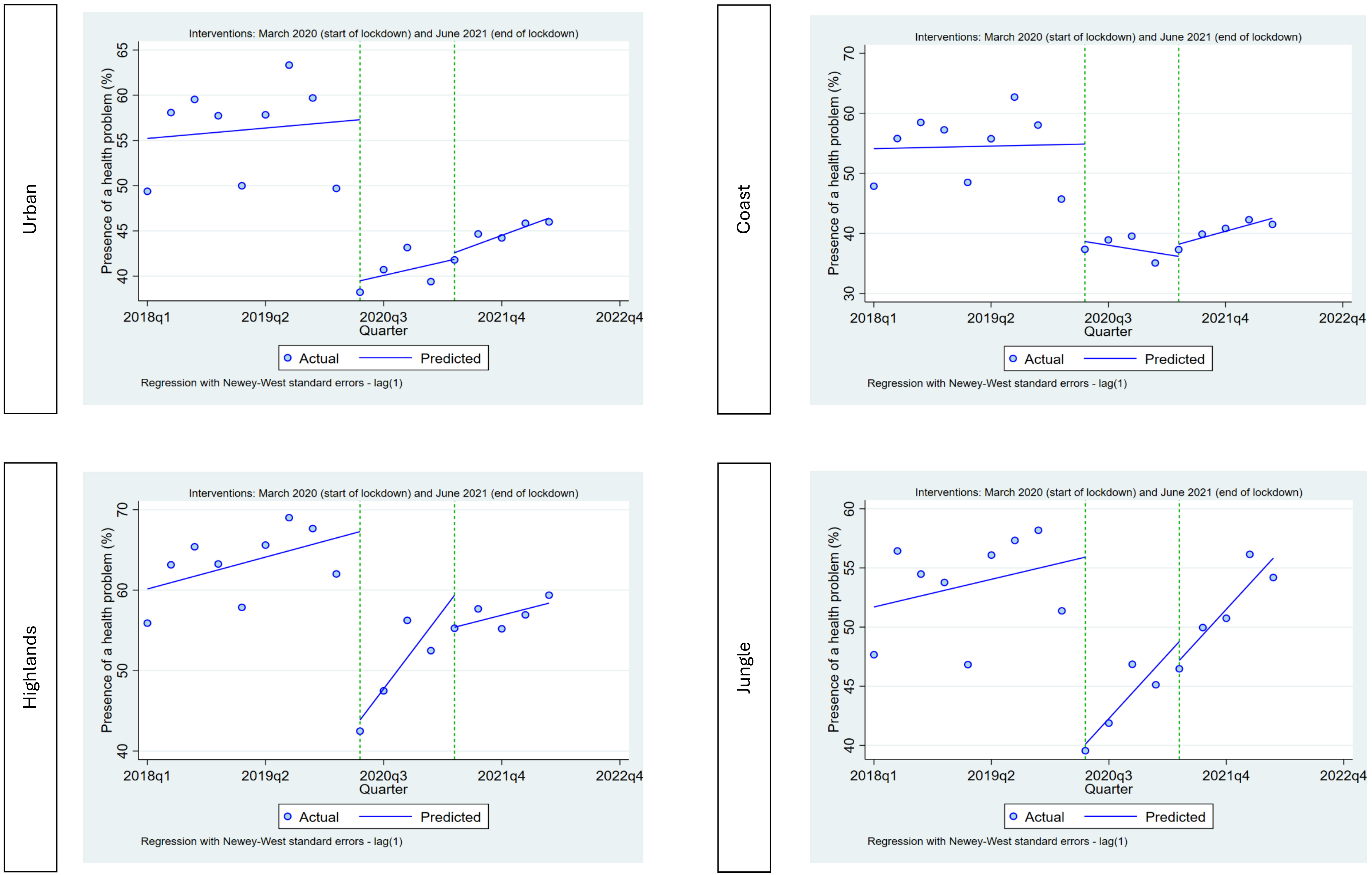
Urban
Interventions: March 2020 (start of lockdown) and June 2021 (end of lockdown)
Presence of a health problem (%)
Quarter
2018q1
2019q2
2020q3
2021q4
2022q4
Actual
Predicted
Regression with Newey-West standard errors - lag(1)
Coast
Interventions: March 2020 (start of lockdown) and June 2021 (end of lockdown)
Presence of a health problem (%)
Quarter
Actual
Predicted
Regression with Newey-West standard errors - lag(1)
Highlands
Interventions: March 2020 (start of lockdown) and June 2021 (end of lockdown)
Presence of a health problem (%)
Quarter
Actual
Predicted
Regression with Newey-West standard errors - lag(1)
Jungle
Interventions: March 2020 (start of lockdown) and June 2021 (end of lockdown)
Presence of a health problem (%)
Quarter
Actual
Predicted
Regression with Newey-West standard errors - lag(1)
