# Supplementary material for: Impact of COVID-19 on unmet needs for healthcare in Peru: an interrupted time series analysis
Source: PLOS Glob Public Health. 2025 Oct 10;5(10):e0005036. doi: 10.1371/journal.pgph.0005036 (PMC12513646; doi:10.1371/journal.pgph.0005036)

S2 Fig. Interrupted time series analysis of the impact of COVID-19 lockdown on unmet need for healthcare, stratified by sociodemographic variables.

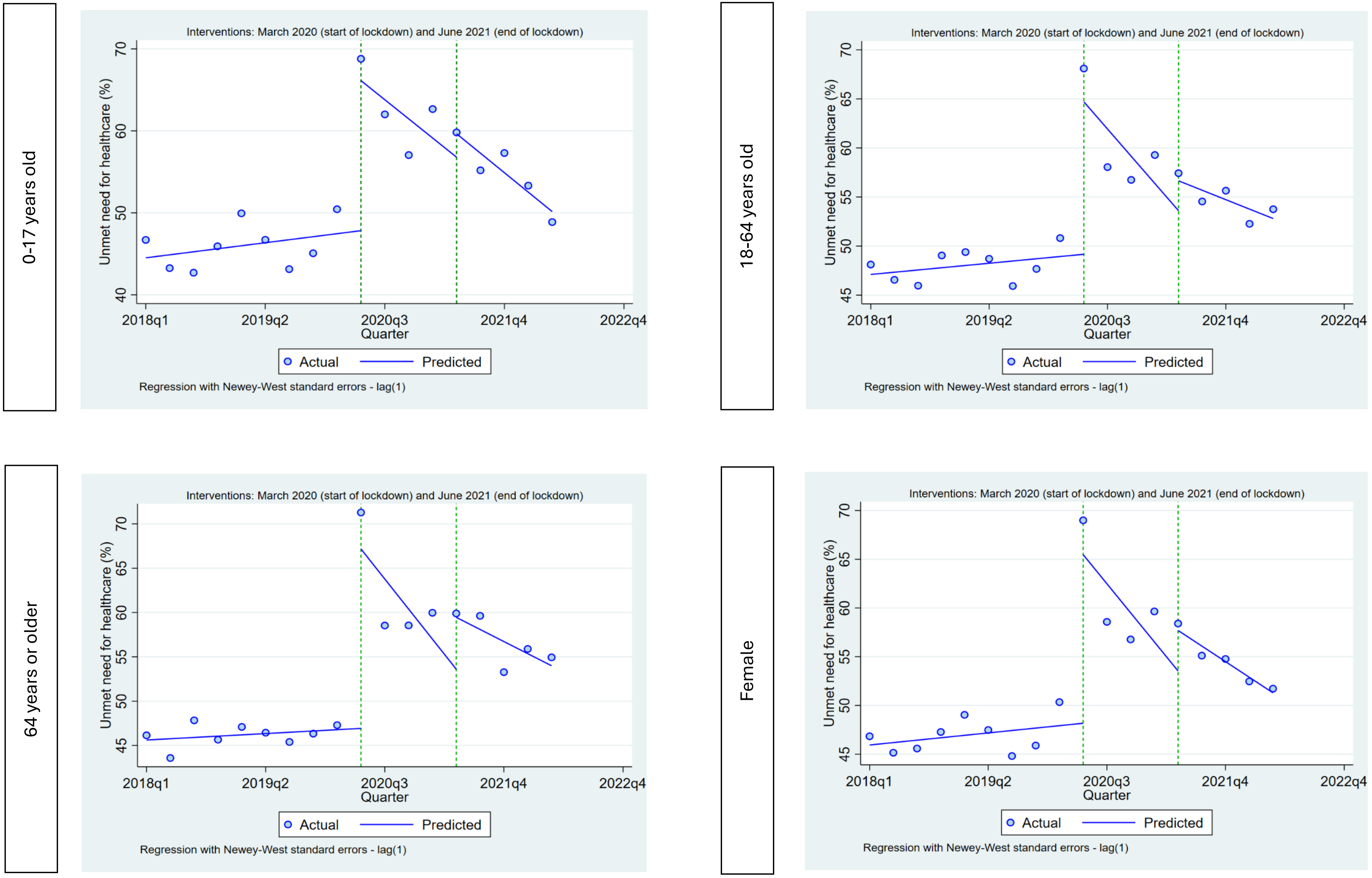

Male

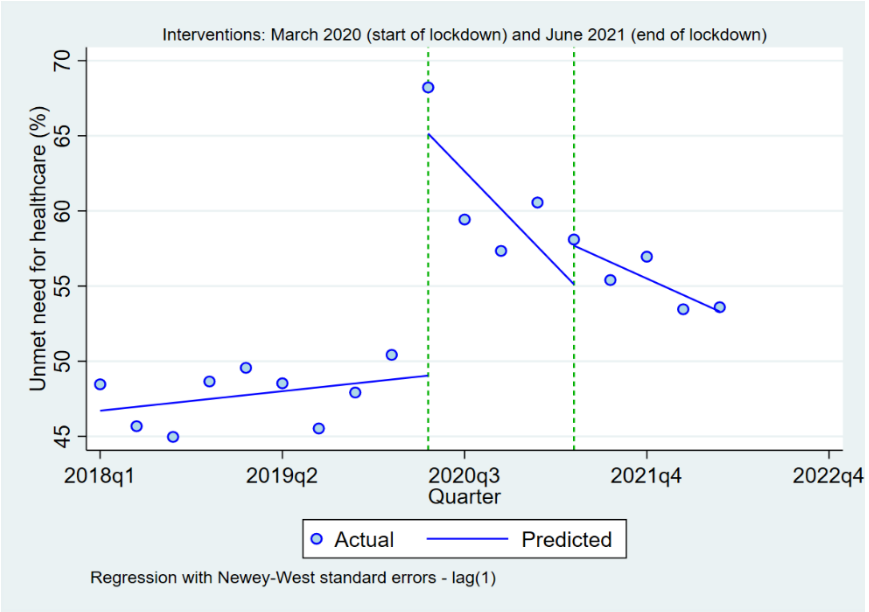

Non-native

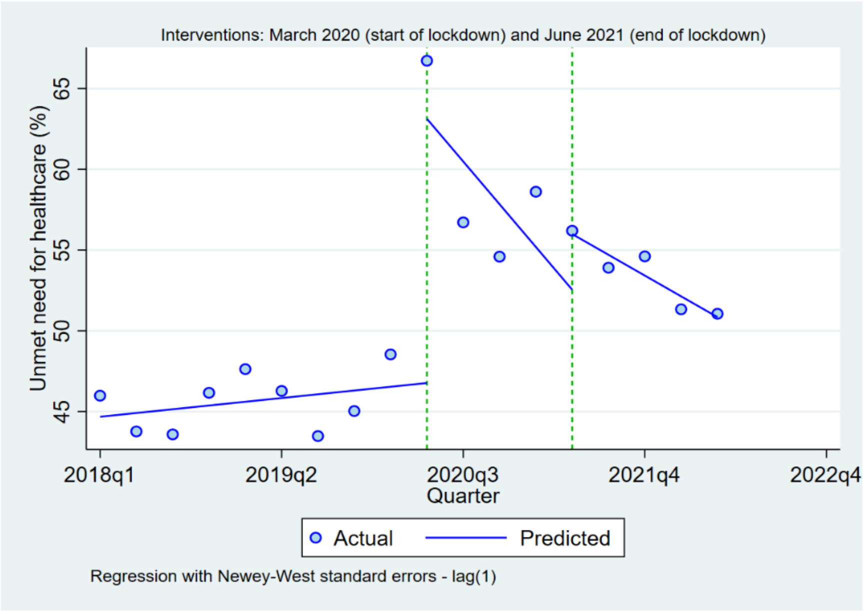

Native

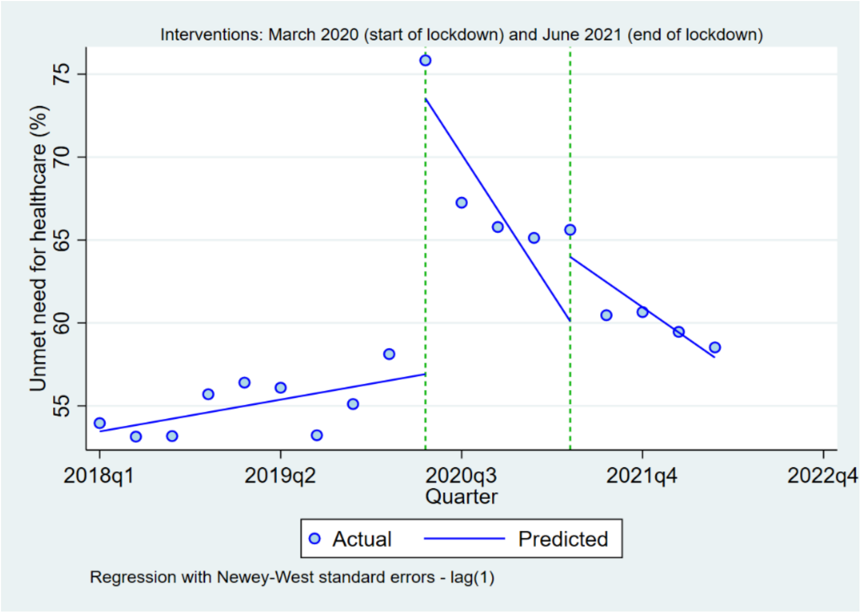

No health insurance

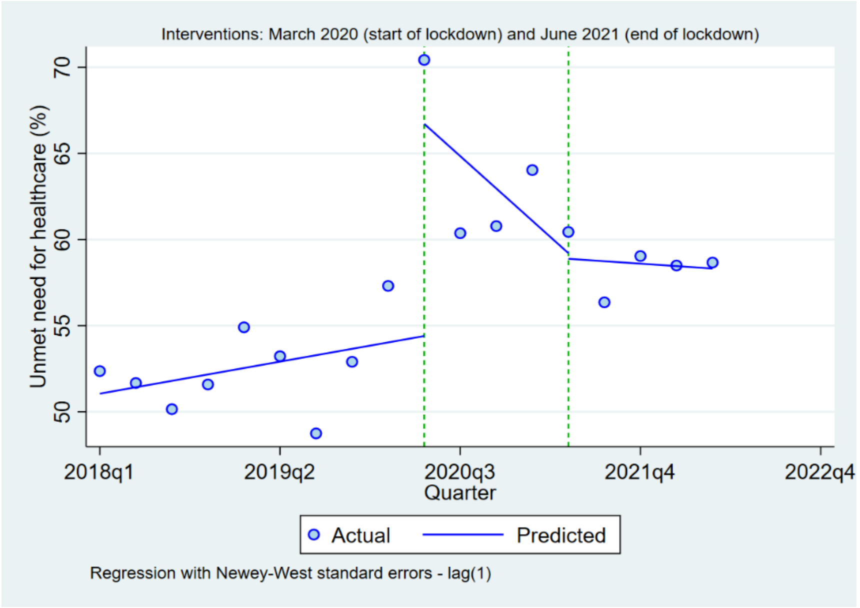

Armes forces and Police

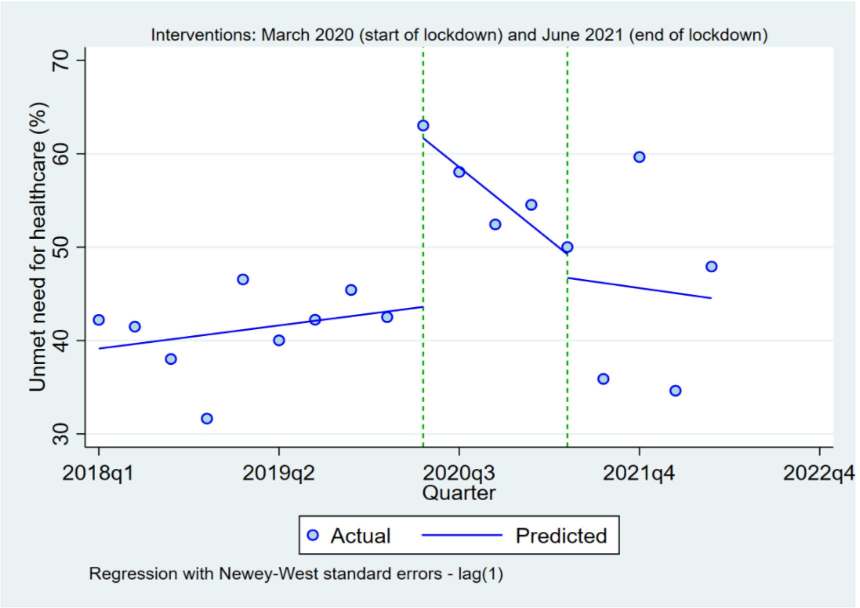

Public health insurance

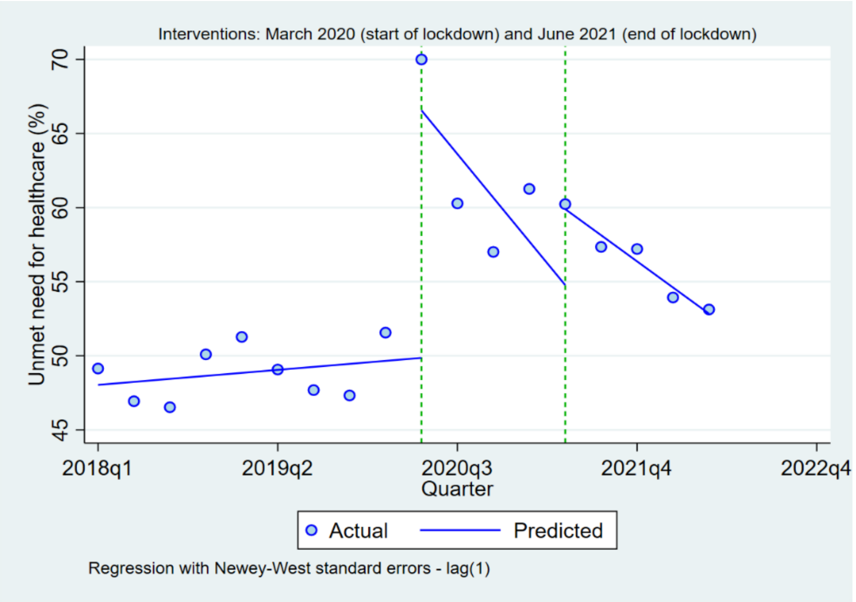

Social health insurance

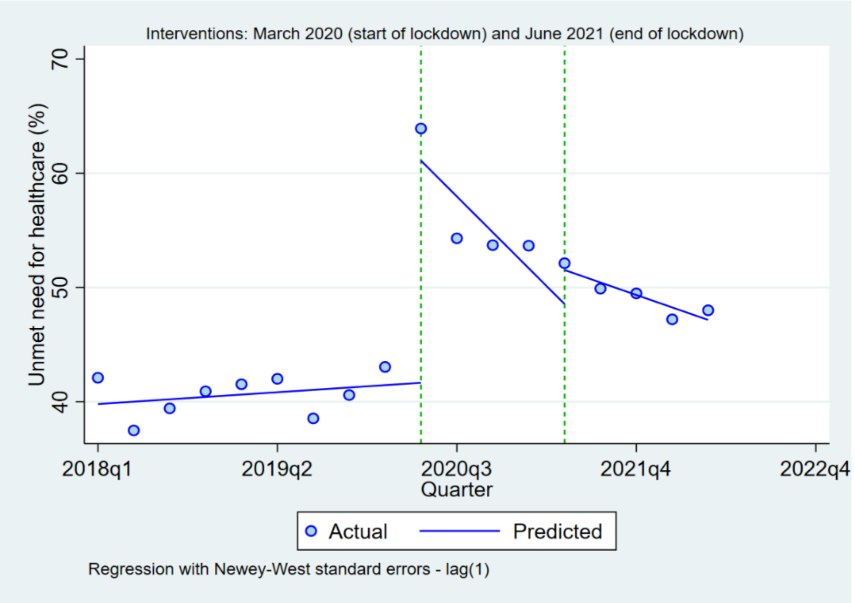

Private insurance

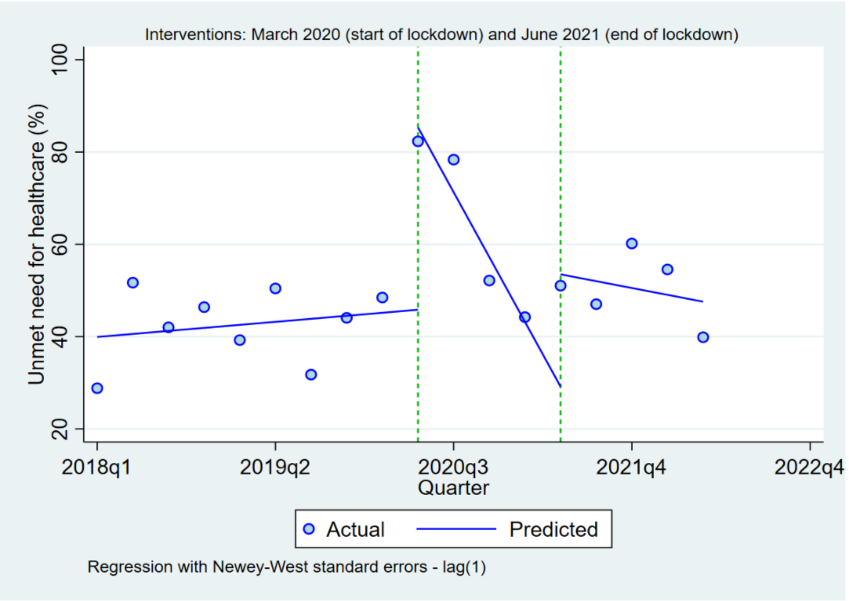

People without disabilities

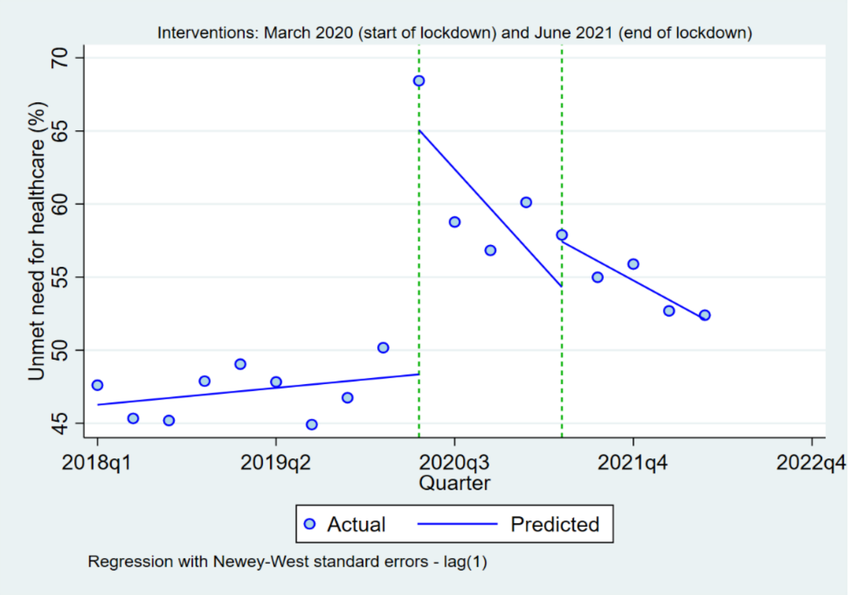

People with disabilities

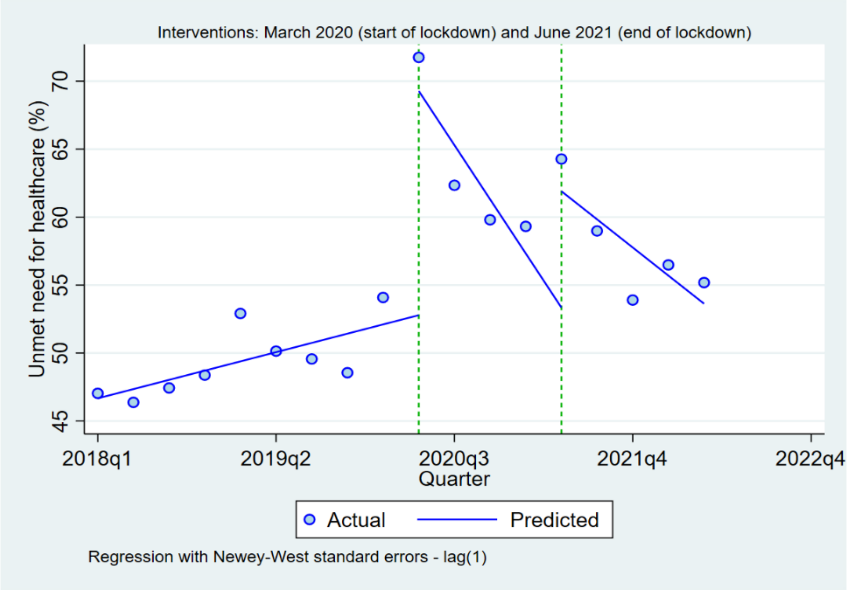

Rural

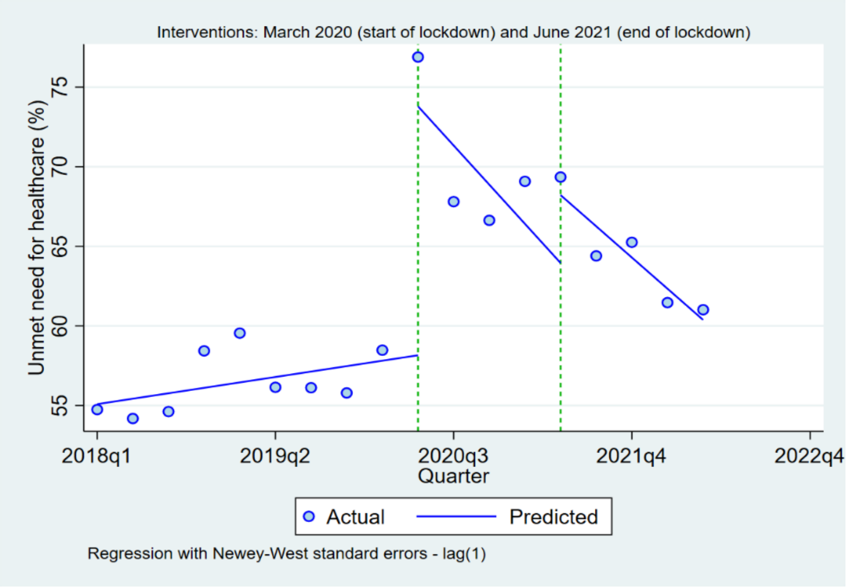

Urban

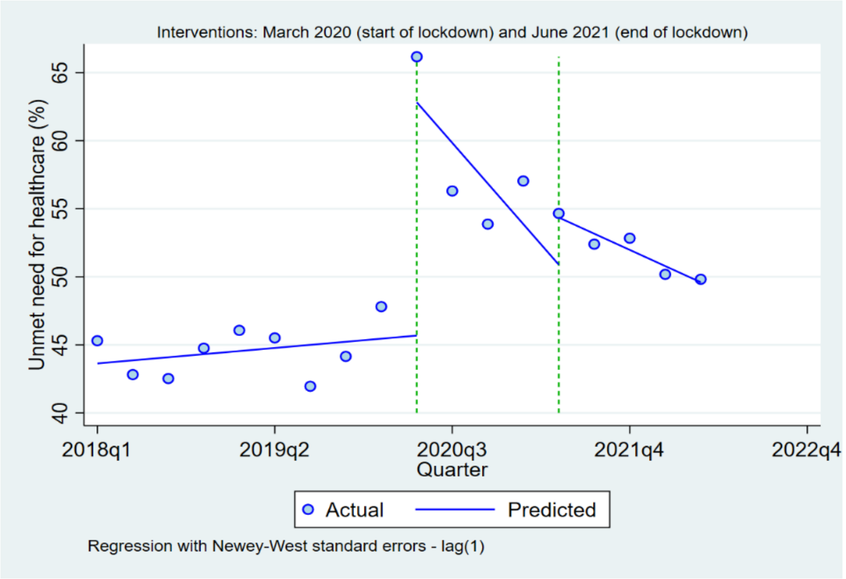

Coast

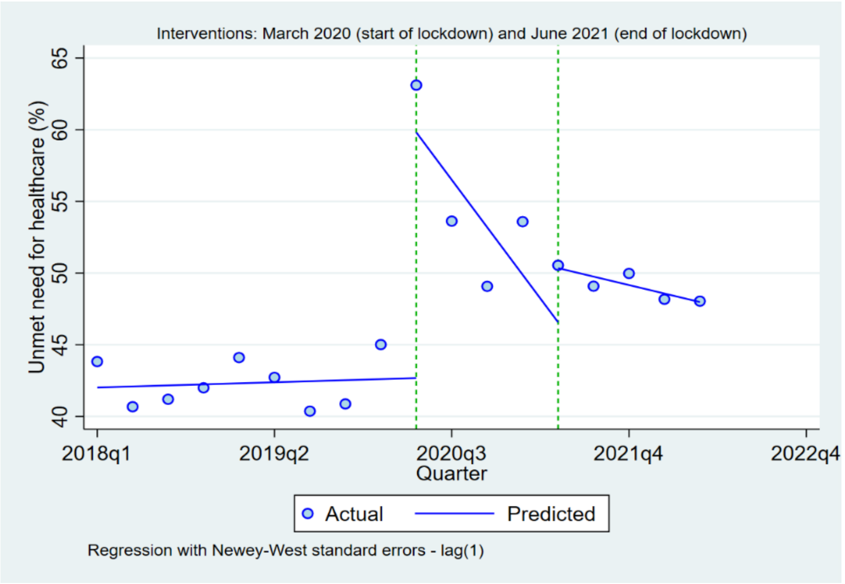

Highlands

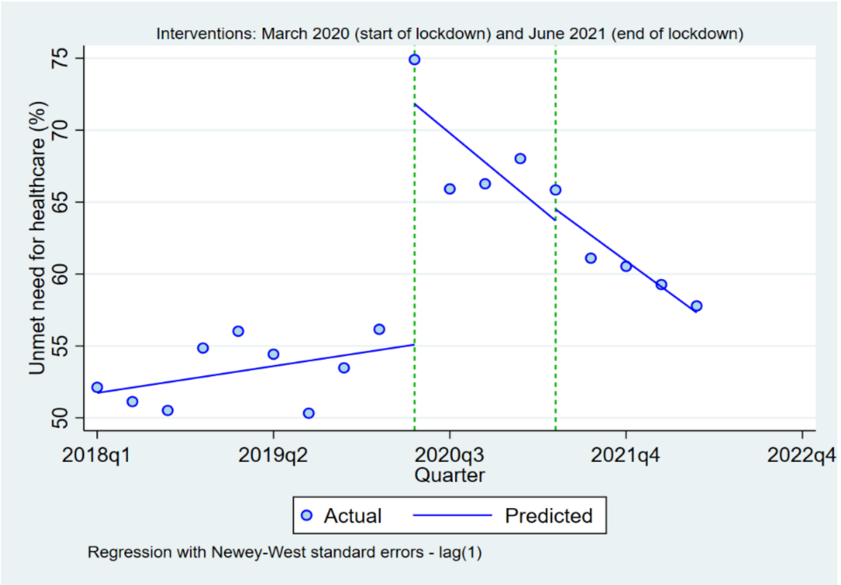

Jungle

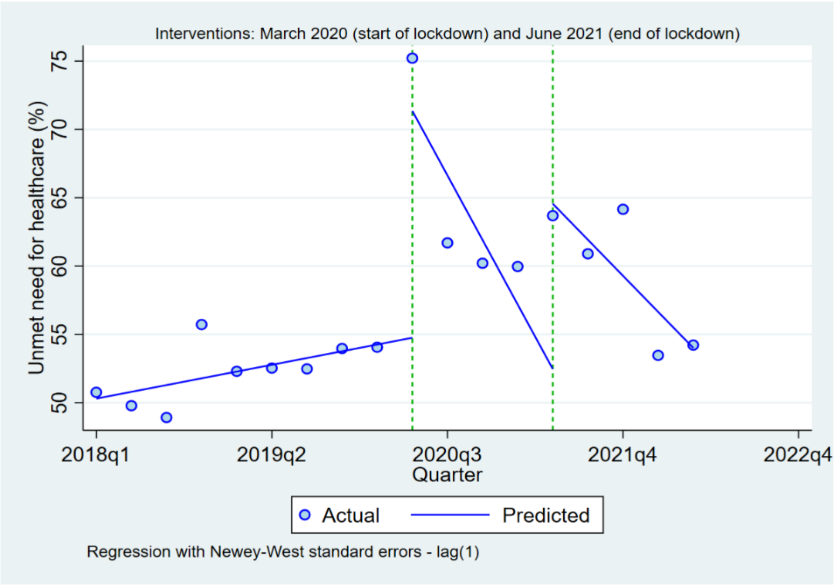

Supplement: S2 Fig — (PDF) [file pgph.0005036.s002.pdf]
