# Supplementary material for: Impact of COVID-19 on unmet needs for healthcare in Peru: an interrupted time series analysis
Source: PLOS Glob Public Health. 2025 Oct 10;5(10):e0005036. doi: 10.1371/journal.pgph.0005036 (PMC12513646; doi:10.1371/journal.pgph.0005036)
Supplement: Attachment — Submitted filename: Response to Reviewers.docx [file pgph.0005036.s004.docx]

**Dear Editor and Reviewers,**

Thank you for your valuable suggestions and remarks. Please find below the point-by-point responses to your comments. Corresponding edits in the main text have been highlighted for your convenience. We hope the present version of the manuscript will be acceptable for publication, and we look forward to your feedback.

**Editor:**

1. Your current Financial Disclosure states, “PENDA project PO8073)”. However, it is missing in your funding information on the submission form. Please indicate by return email the full and correct funding information for your study and confirm the order in which funding contributions should appear. Please be sure to indicate whether the funders played any role in the study design, data collection and analysis, decision to publish, or preparation of the manuscript.

**Response:** Thank you for the observation.The required information has been included.

**Changes:** “*The research was funded by the UK Foreign, Commonwealth and Development Office under 2 grants (PENDA project - PO8073; COVID Collective - 102866EH). Part of LMB’s time and Open Access is covered by the Arts & Humanities Research Council (AH/X009580/1).*”

**Reviewer #1**

Methods:

1. Does the parametrization include a vector of confounding covariates?

**Response**: Thank you for the observation. The analysis focused exclusively on the outcome variable (unmet need for healthcare), aiming to highlight changes that occurred before, during, and after lockdown without attempting to establish causality. Since causality was not considered and only the outcome variable was included in the model, no confounding variables were included (those associated with both the exposure and the outcome and not in the causal pathway). For this reason, we thought it relevant to observe whether the measured outcome might vary across different population characteristics. Thus, we conducted a stratified analysis based on various population characteristics, such as age group, gender, health insurance coverage, area of residence, disability status, Indigenous status, and natural region. This approach allowed us to measure whether Indigenous people, individuals with disabilities, or those with other characteristics experienced similar or different changes compared to their counterparts. This pattern has also been observed in other studies: <https://bmcpublichealth.biomedcentral.com/articles/10.1186/s12889-024-20452-6>, <https://pmc.ncbi.nlm.nih.gov/articles/PMC11487949/>, <https://bmcmedicine.biomedcentral.com/articles/10.1186/s12916-024-03664-w>, <https://pmc.ncbi.nlm.nih.gov/articles/PMC10682841/#bib9>, <https://pubmed.ncbi.nlm.nih.gov/39439763/>, <https://pmc.ncbi.nlm.nih.gov/articles/PMC10864310/>

**Changes:** none

2. The current parametrization lacks addressing serial correlation in the error terms. Newey West variance estimator is advised.

**Response**: Thank you for the observation. All details of the interrupted time series analysis have been included. In this analysis, we used Newey-West standard errors to correct for potential autocorrelation in the model.

**Changes:** “*Newey-West standard errors were applied to correct for autocorrelation [26]. To determine the appropriate number of lags for a model that accurately reflects the correct autocorrelation structure, the actest post-estimation command was used [26]. In all interrupted time series analyses, a lag of order one was used, as the p-value >0.05, suggesting that the null hypothesis of no autocorrelation among the data cannot be rejected.*”

Results:

3. Figures are not available for review. It would be great to display a visualization of the empirical distribution of the outcomes, the fitted and predicted regression lines.

**Response**: Thank you for the observation. We have included figures of the interrupted time series analysis for better data visualization. The two main figures are presented as a panel figure within the manuscript, while the figures for the interrupted time series analysis conducted for each stratification variable category are in the Supporting Information.

**Changes**: We included Fig. 1, Fig. S1, and Fig. S2 in the Supporting Information.

4. The coefficients table look a bit odd. The binary variables report two coefficients without setting a reference level.

**Response**: Thank you for the observation. The stratified analysis was conducted in two phases: i) In the first phase, the percentage of the outcome (unmet need for healthcare) was calculated for each category of the stratification variables across all quarters included in the study. This calculation was performed using the following command: *svy: mean outcome, over(stratification variable)*; and ii) Using the data on the percentage of the outcome in each period, an interrupted time series analysis was then conducted with the itsa command. This analysis was performed for each category of the stratification variables, treating them as subpopulations rather than as independent variables. For example, for the variable “disability status,” the percentage of the outcome was calculated for individuals with and without disabilities in each quarter included in the study. Then, a separate interrupted time series analysis was conducted for individuals with disabilities and for those without. Under this approach, the stratification variables were not treated as independent variables within the model, as the proposed analysis did not aim to establish causality but rather to observe changes in the outcome before, during, and after lockdown across different population characteristics.

This approach has been observed in various studies: <https://bmcpublichealth.biomedcentral.com/articles/10.1186/s12889-024-20452-6>, <https://pmc.ncbi.nlm.nih.gov/articles/PMC11487949/>, <https://bmcmedicine.biomedcentral.com/articles/10.1186/s12916-024-03664-w>, <https://pmc.ncbi.nlm.nih.gov/articles/PMC10682841/#bib9>, <https://pubmed.ncbi.nlm.nih.gov/39439763/>, <https://pmc.ncbi.nlm.nih.gov/articles/PMC10864310/>

**Changes:** none

Discussion:

5. This section should be enhanced by connecting the findings with the real impact in the Peruvian Healthcare system. For now, that connection is lacking.

**Response**: Thank you for the observation. We have included more discussion on the impact ot the Peruvian healthcare system throughout.

**Changes**: Various additions throughout the discussion.

**Reviewer #2:**

This paper provides an exceptional analysis of the impact of COVID-19 safety measures in Peru. It presents valuable data from a low-middle income country (LMIC), a rarity in current literature. The primary aim of this study was to assess the impact of COVID-19 on unmet healthcare needs across Peru. This analysis will be highly useful for preparing for future pandemics in similar contexts.

1. The study focuses on understanding unmet health needs before and after lockdowns. An interrupted time series analysis was conducted using the National Household Survey on Living Conditions and Poverty (ENAHO) data from 2018 to 2022. ENAHO is a population-based survey managed by the National Institute of Statistics and Informatics of Peru.

**Response**: Thank you for the observation. The interrupted time series analysis we conducted indeed aims to assess changes in unmet health needs around the COVID-19 lockdown periods. Using data from ENAHO, a comprehensive, population-based survey conducted by the National Institute of Statistics and Informatics of Peru, allows us to examine this transition over the span of 2018 to 2022. This approach provides robust insights by using an established dataset that reflects national health and living conditions across these key years.

2. The use of data collected through both face-to-face and telephone interviews with trained interviewers is a major strength of this study. The total and annual sample sizes are strong, and the response rate is impressive, adding reliability to the findings.

**Response**: Thank you for the observation. We agree that the use of data collected through both face-to-face and telephone interviews conducted by trained interviewers significantly enhances the robustness of our results.

3. The dependent variables are well-defined, and the analysis is stratified based on disability status and indigenous status. Other variables, such as age, gender, health insurance coverage, and geographical regions, are also included and thoroughly described, showcasing the comprehensiveness of the data.

**Response**: Thank you for the observation. This comprehensive approach not only enriches the data but also enhances the robustness of our analysis, allowing for a more nuanced understanding of the factors influencing health outcomes in the study population.

4. Statistical analyses are meticulously detailed and disaggregated. Although I am not an expert in the mathematical modeling of interrupted time series, the methodology appears sound and consistent with other high-quality research.

**Response**: Thank you for the observation. We appreciate your acknowledgment of the meticulous detail and disaggregation in our statistical analyses.

5. The results are presented clearly, and the discussion is compelling. The use of graphical presentations for the interrupted time series could further enhance the visual understanding of the findings.

**Response**: Thank you for the observation. We have included figures of the interrupted time series analysis for better data visualization. The two main figures are presented as a panel figure within the manuscript, while the figures for the interrupted time series analysis conducted for each stratification variable category are in the Supporting Information.

**Changes**: We included Fig. 1, Fig. S1, and Fig. S2 in the Supporting Information.

6. The limitations are transparently presented and appropriately addressed.

**Response**: Thank you for the observation. We believe that openly addressing these limitations is crucial for understanding the context of our findings and for guiding future research.

7. Overall, I find this manuscript exceptionally informative and well-written. It merits publication.

**Response**: Thank you very much for your positive feedback and endorsement of our manuscript.
