# Supplementary material for: Impact of COVID-19 on unmet needs for healthcare in Peru: an interrupted time series analysis
Source: PLOS Glob Public Health. 2025 Oct 10;5(10):e0005036. doi: 10.1371/journal.pgph.0005036 (PMC12513646; doi:10.1371/journal.pgph.0005036)
Supplement: Attachment — Submitted filename: Response to Reviewers (3).docx [file pgph.0005036.s006.docx]

**Dear Editor and Reviewers,**

Thank you for your valuable suggestions and remarks. Please find below the point-by-point responses to your comments. Corresponding edits in the main text have been highlighted for your convenience. We hope the present version of the manuscript will be acceptable for publication, and we look forward to your feedback.

**Editor:**

1. Please review your reference list to ensure that it is complete and correct. If you have cited papers that have been retracted, please include the rationale for doing so in the manuscript text, or remove these references and replace them with relevant current references. Any changes to the reference list should be mentioned in the rebuttal letter that accompanies your revised manuscript. If you need to cite a retracted article, indicate the article’s retracted status in the References list and also include a citation and full reference for the retraction notice.

**Response:** We have verified the references and to our knowledge none of the included references have been retracted. If the reviewers/Editors have concerns about a specific reference, please do let us know.

2. Clarify measurement of unmet need: The operationalization of "unmet healthcare need" could be interpreted variably. A clearer explanation and possible limitations (e.g., care sought in non-biomedical facilities) should be emphasized in the limitations section.

**Response:** We have now substantially changed the limitations section, and added more clarity on the construction of the unmet need variable in the methods.

3. Limitations Expansion: While some limitations are acknowledged, additional discussion around the effect of changes in data collection mode (e.g., shift to phone interviews) and potential reporting bias during lockdown periods would be valuable.

**Response:** Thank you for the observation. The limitations section now reads as follows: *“**Another limitation is that the transition from face-to-face interviews to telephone interviews during the COVID-19 lockdown period may have introduced reporting bias. Although response rates were high for all reporting periods – including periods with telephone interviews – the quality of data collection may have been more limited with phone interviews compared to face-to-face. These limitations may reduce the comparability of data across time periods and could contribute to measurement variability in the estimated prevalence of* *unmet need for healthcare.”*

**Reviewer #3**

1. Write the abstract in the correct order (background, objectives, methods, results, and conclusion).

**Changes:** The abstract section now reads as follows: “*The COVID-19 pandemic affected people’s health and access to healthcare worldwide. Still, little is known about how distinct phases of the pandemic – namely the lockdown period and the period directly after lockdown – impacted these outcomes, and on if there were differences in these impacts among different segments of the Peruvian population. The aim of this study was to examine the impact of COVID-19 on unmet needs for healthcare across Peru. Using an interrupted time series design, this study analyzed quarterly data from Peru's National Household Survey on Living Conditions and Poverty (ENAHO) from 2018 to 2022. Outcome variables included people reporting having a health problem in the last 4 weeks and, amongst those reporting a problem, whether they had needed and sought care. These outcomes were stratified by demographic characteristics (i.e. by age group, gender, disability, indigenous status, regions and rural/urban). Step and trend changes in outcomes during and directly after lockdown periods were compared to the pre-pandemic period. Overall, the prevalence of health problems decreased by 18.4 percentage points (pp) during the lockdown period compared to pre-pandemic levels, although unmet healthcare needs increased by 16.7pp. Once lockdowns were removed, prevalence of health problems and access to healthcare increased slightly, but remained below pre-pandemic levels. Certain groups, namely people with disabilities, indigenous people and people in rural areas, had persistently worse health and poorer access to care. Persistent disparities in unmet healthcare needs highlight the necessity of targeted interventions to address systemic barriers faced by vulnerable populations and ensure continuity of care during health crises.*”

2. Make table 3 more understandable or merge with table 2 if possible.

Thank you for the observation. We considered that Table 3 follows a format similar to that of other articles published in PLOS Global Public Health, which include one table presenting proportions and a separate table exclusively reporting the results of the time series analysis. The following articles are examples of this format: <https://journals.plos.org/globalpublichealth/article?id=10.1371/journal.pgph.0000372>, <https://journals.plos.org/globalpublichealth/article?id=10.1371/journal.pgph.0003351>. Moreover, the importance of reporting both the step and trend change lies in their ability to capture two distinct aspects of the lockdown's impact. The step change reflects the immediate effects, while the trend change indicates a modification in the trajectory of the outcome over time. This reporting approach is observed in other studies: <https://bmcpublichealth.biomedcentral.com/articles/10.1186/s12889-024-21256-4>, <https://www.nature.com/articles/s41598-022-15569-w>, <https://pmc.ncbi.nlm.nih.gov/articles/PMC11726930/>, <https://bmcinfectdis.biomedcentral.com/articles/10.1186/s12879-025-10444-1>.

Nonetheless, we have revised the titles of Tables 3 and 5 to improve clarity.

3. The discussion is good, but the conclusion looks like a recommendation.

**Response**: The conclusion section now reads as follows: *“**This research carries important implications for preparedness for future pandemics and other public health crises, as well as for the routine operation of health systems in Peru and elsewhere. It is clear that both pre- and during COVID-19, certain groups (e.g., people with disabilities, Indigenous people and people in rural and remote areas) experienced worse health outcomes and poorer access to care. As such, more targeted approaches are needed to address the persistent barriers faced by these groups, such as through improving the geographic availability, affordability and accessibility of health services. Further, the needs of specific groups at risk of exclusion must be considered in pandemic preparedness planning in Peru and other contexts.”*
